# Structural insight into the DNMT1 reaction cycle by cryo-electron microscopy

**DOI:** 10.1371/journal.pone.0307850

**Published:** 2024-09-03

**Authors:** Inessa De, Jonas Weidenhausen, Nestor Concha, Christoph W. Müller

**Affiliations:** 1 European Molecular Biology Laboratory (EMBL), Structural and Computational Biology Unit, Heidelberg, Germany; 2 GlaxoSmithKline, Collegeville, PA, United States of America; Universita degli Studi Gabriele d’Annunzio Chieti e Pescara, ITALY

## Abstract

DNMT1 is an essential DNA methyltransferase that catalyzes the transfer of methyl groups to CpG islands in DNA and generates a prominent epigenetic mark. The catalytic activity of DNMT1 relies on its conformational plasticity and ability to change conformation from an auto-inhibited to an activated state. Here, we present four cryo-EM reconstructions of apo DNMT1 and DNTM1: non-productive DNA, DNTM1: H3Ub2-peptide, DNTM1: productive DNA complexes. Our structures demonstrate the flexibility of DNMT1’s N-terminal regulatory domains during the transition from an apo ‘auto-inhibited’ to a DNA-bound ‘non-productive’ and finally a DNA-bound ‘productive’ state of DNMT1. Furthermore, we address the regulation of DNMT1’s methyltransferase activity by a DNMT1-selective small-molecule inhibitor and ubiquitinated histone H3. We observe that DNMT1 binds DNA in a ‘non-productive’ state despite the presence of the inhibitor and present the cryo-EM reconstruction of full-length DNMT1 in complex with a di-ubiquitinated H3 peptide analogue. Taken together, our results provide structural insights into the reaction cycle of DNMT1.

## Introduction

DNA methylation in eukaryotes involves the transfer of a methyl group from S-adenosylmethionine to cytosine’s C5 by DNA methyltransferase enzymes, DNMTs. The 5-methylcytosine (5mC) product occurs most commonly on cytosines that precede a guanine nucleotide in CpG dinucleotides and these methyl marks are commonly found within clusters of symmetrical CpG dinucleotide sequences on double stranded DNA known as CpG islands [1]. In adult somatic cells CpG islands have a variety of methylation states–ranging from unmethylated to hypermethylated regions [2]. The presence of 5mC is a key epigenetic signal for the regulation of gene expression. In differentiated cells, a unique and stable 5mC-DNA methylation pattern is responsible for cell-specific gene transcription, but in many cancer types an altered DNA methylation pattern appears to be responsible for the inactivation of tumor-suppressor genes, for promoting genomic instability and for cell transformation [3–5]. For this reason, pharmacological modulation of DNA methylation is being studied as a means to treat various forms of cancer [6].

DNMT1, DNMT3A and DNMT3B (DNMT3A/B) are the canonical 5mC DNMTs in human cells. They share a similar architecture with a large N-terminal regulatory region and a C-terminal catalytic region [7, 8]. Functionally, they complement each other to produce DNA methylation in two stages catalyzed by different DNMTs: DNMT3A/B introduce the initial methyl groups to unmethylated cytosines and establish a methylation pattern that must be maintained during DNA replication. Whereas DNMT1 maintains the DNA methylation pattern on newly replicated DNA [9, 10] by localizing to the replication fork where newly synthesized hemi-methylated DNA is formed [11, 12].

**Fig 1:**
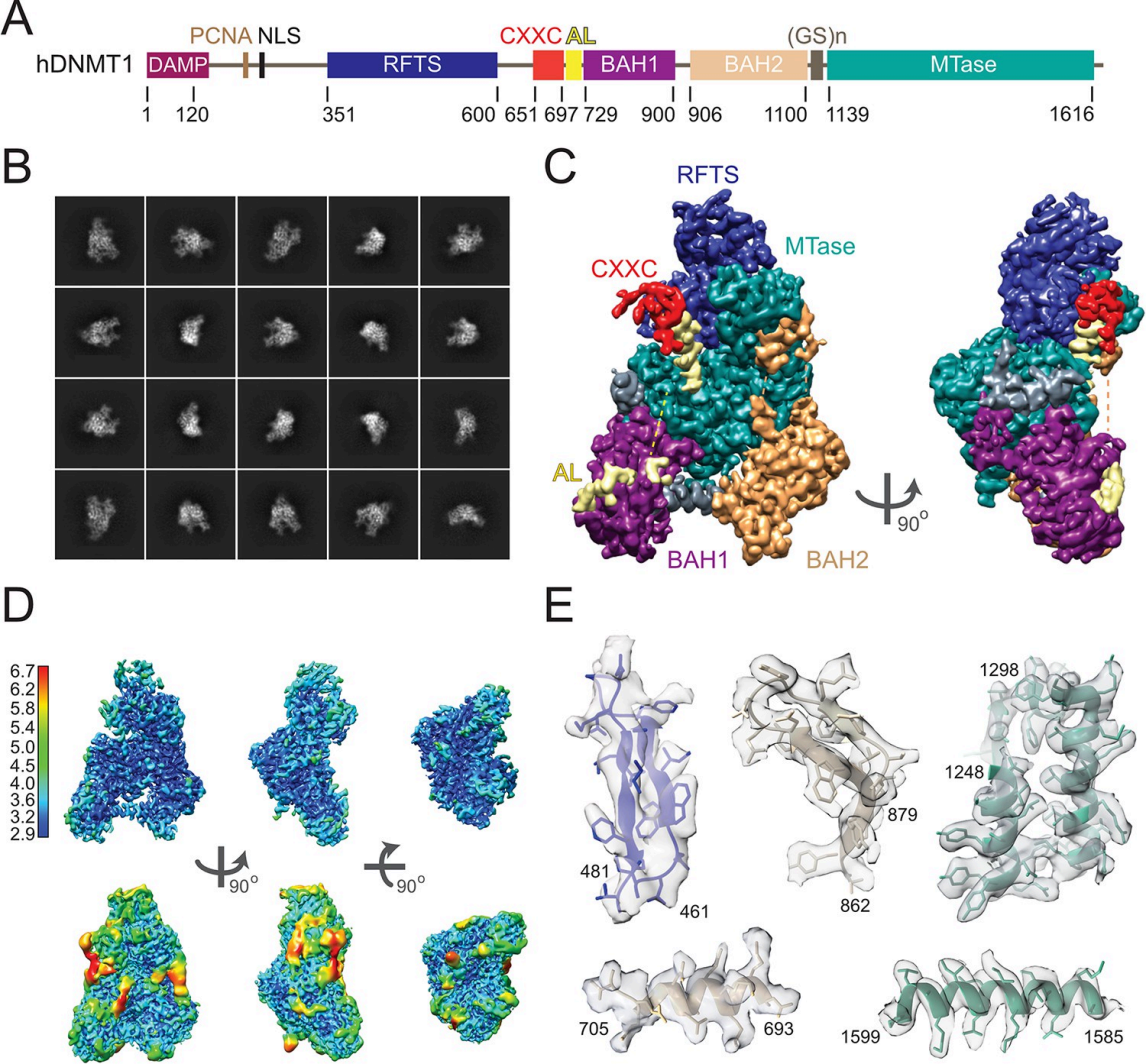
Single particle cryo-EM reconstruction of apo DNMT1. (A) Domain organization of human DNMT1. (B) Representative 2D class averages calculated from micrographs showing different particle orientations. (C) Cryo-EM density map of apo DNMT1 in front and side views. Domains are color-coded as in (A). (D) Cryo-EM density map colored according to the resolution distribution at higher (upper panel) and lower (lower panel) contour levels. The resolution (Å) is color-coded as indicated on the bar. (E) Representative regions of the cryo-EM map with the crystal structure of apo human DNMT1 (PDB 4WXX) fitted in.

DNMT1 is the largest of the DNMTs with its methyltransferase catalytic (MTase) domain preceded by at least five regulatory domains: DMAP1-binding domain (DNA methyltransferase 1-associated protein 1), RFTS domain (Replication Focus Targeting Sequence), CXXC zinc finger domain, and two BAH domains (Bromo Adjacent Homology) [13] (Fig 1A). At rest, DNMT1 is auto-inhibited, most likely by a number of interactions between the catalytic domain and the RFTS and CXXC domains [14]. The RFTS domain occupies the DNMT1 catalytic pocket [15, 16] and prevents the hemi-methylated DNA substrate from binding. In addition, the CXXC-BAH1 linker blocks the access of unmethylated DNA to the binding pocket to prevent *de novo* methylation. In the presence of hemi-methylated DNA, the CXXC domain binds to unmethylated CpG sequences [14, 17]. Additionally, a critical regulatory step is mediated by the interaction of DNMT1 with the E3 ubiquitin ligase, UHRF1 (ubiquitin-like, containing PHD and RING finger domain 1) [18]. UHRF1 binds to DNMT1 during DNA replication [19] and the functions of these two proteins are so essential that the knockout of either protein leads to embryonic lethality [20]. UHRF1 also binds methylated cytosines via its SRA domain (SET- and RING-associated DNA-binding domain) and flips the methylated base out of the DNA helix [21]. In this way, it provides DNMT1 access to its substrate. Additionally, UHRF1 ubiquitinates histone H3 on lysine residues K14, K18 and/or K23 and the di-ubiquitinated histone H3 binds the RFTS domain of DNMT1 [22, 23]. The di-ubiquitinated histone H3 (H3-K18Ub/K23Ub) binds the RTF domain approximately 100 times stronger than unmodified histone H3 peptide [22]. Thus, the activation of DNMT1 involves, at least the following steps: (i) interaction with UHRF1 and recognition of di-ubiquitinated histone H3, (ii) recognition of the hemi-methylated-DNA by the SAR domain of UHRF1, (iii) shift of the RFTS domain away from the catalytic site, and (iv) shift of the CXXC-BAH1 domains away from the active site, making it available and accessible to the DNA substrate. Furthermore, the rearrangement of the CXXC domain in the presence of the H3Ub2: DNA ternary complex is coupled to the conformational changes of the recognition helix (residues 1236–1259) with the phenylalanine residues F631 and F632 recognizing hemi-methylated DNA [24].

In this manuscript, we set out to further understand the dynamics of the domain rearrangements during the activation of DNMT1. We show how the structure of full-length DNMT1 changes during the reaction cycle from an apo ‘auto-inhibited’ state to DNA-bound ‘non-productive’ state (with inhibitor) to DNA-bound ‘productive’ state (with H3Ub2-peptide analogue). Recently, a new class of DNMT1-selective non-nucleoside dicyanopyridine inhibitors and their mode of action have been described [6, 25]. We show that full-length DNMT1 is stalled in the ‘non-productive’ DNA-bound state in the presence of one of the related inhibitors GSK3852279B (related structure: PDB ID 7SFF).

## Material and methods

### Recombinant DNMT1 and DNA oligos

Recombinant human full-length DNMT1 was expressed by Genscript and purified by GSK with the following steps: Expression of DNMT1 with N-terminal tag containing Flag-6xHis-TEV protease cleavage site was performed using the baculovirus/*Sf*9 insect cell expression system. Purification to >85% purity was performed by affinity purification with Ni-NTA column followed by a Superdex 200 size exclusion chromatography. The DNMT1 fractions were pooled and concentrated to 15.9 mg/ml as measured by absorbance at 280 nm (molecular weight = 189.417 kDa, molar absorption coefficient ε_280nm_ = 159980 M^-1^cm^-1^) and frozen at -80°C in 20 mM Tris, 200 mM NaCl, 5% glycerol, 1 mM DTT, pH 7.5. The identity of the purified protein was confirmed by mass spectrometry with 81% sequence coverage.

To reconstitute DNMT1 complexes with DNA, a 26 base pair duplex DNA containing a single central hemi-methylated cytosine [mC] and with or without 5-fluorodeoxycytosine (5-fC) was synthesized by the Keck Oligonucleotide Synthesis Resource, Yale University. Hemi-methylation (mC) of DNA is needed to be recognized as a DNMT1 substrate with the opposite 5-fC forming an irreversible, covalent link with DNMT1 after its enzymatic activity at this site [9]. The parental strand was 5’-TGGAGTCGAGGC[mC]GCCTGCGCAGGAC-3’, the target strands with and without 5-fC were 5’-GTCCTGCGCAGG[fC]GGCCTCGACTCCA-3’ and 5’-GTCCTGCGCAGGCGGCCTCGACTCCA-3’. Parental and target strands of DNA were hybridized by mixing equimolar amounts and incubating at 95°C for 3 min, then transferring onto ice and adding the same volume of hybridization buffer (40 mM HEPES pH 7.5, 24 mM MgCl_2_, 200 mM NaCl, 20 mM DTT) to it. Additionally, a hemi-methylated double strand DNA without 5-fC on the target strand was hybridized in the same way. Duplex DNA containing 5-fC (5FDNA) was used to reconstitute the DNMT1: productive DNA complex (DNMT1:5FDNA: H3Ub2-peptide) where 5FDNA forms a covalent bond with DNMT1 and for the DNMT1 methylation assay. Duplex DNA without 5-fC was used to reconstitute the DNMT1: non-productive DNA complex (DNMT1: DNA: GSK3852279B) and to test binding in an EMSA assay.

The GSK3852279B compound was synthesized and provided by GSK.

### Preparation of the H3-K18Ub/K23Ub peptide analogue

The di-ubiquitinated histone H3 peptide analogue (from now on referred to as H3Ub2 peptide) was prepared as previously described [22]. In order to attach ubiquitin via disulfide bonds, K18 and K23 of the H3 peptide were replaced with cysteine (ARTKQTARKSTGGKAPRCQLATCAARKSAPATGGVKW). The peptide was synthesized by Peptide Specialty Laboratories GmbH, Heidelberg, Germany. To be able to form a disulfide bond with the peptide, G76 of ubiquitin (original plasmid with human wildtype ubiquitin was acquired from Addgene, plasmid #12647 –called pET15::Ub) was mutated to cysteine using site-directed mutagenesis according to the manufacturer’s protocols (Q5 site-directed mutagenesis kit, New England Biolabs, #E0554S) with the two DNA oligos GTCTTAAGACTAAGAGGTTGCTGATGAGGTACCCCATGG and CCATGGGGTACCTCATCAGCAACCTCTTAGTCTTAAGAC (mutated site underlined). The G76C ubiquitin mutant construct cloned in pET15 vector (pET15::Ub-G76C) was overexpressed in *E*. *coli* BL21 (DE3) pLysS [26]. Cells were grown at 37°C in 2x YT medium containing 100 μg/ml ampicillin and 33 μg/ml chloramphenicol until they reached an OD600 of 0.6. Expression was induced by adding 0.4 mM IPTG and cells were incubated for 4 h. Cells were lysed by sonication (3 min at 4°C with a duty cycle of 30% and output power of 3 using a Sonifier 250 Ultrasonic Homogeniser, Branson) in buffer containing 50 mM sodium acetate, pH 4.5, 5 mM DTT and a protease inhibitor cocktail (Roche). The lysate was cleared by centrifugation at 235,000 g for 60 min at 4°C (Type 45 Ti rotor, Beckman Coulter). The soluble fraction was then heated at 85°C for 10 min and the remaining precipitates were removed by centrifugation at 235,000 g for 60 min at 4°C (Type 45 Ti rotor, Beckman Coulter). The soluble proteins were further purified on a cation-exchange column (10 mL HiTrap HP SP, GE Healthcare) at 2 ml/min with a gradient from 0% buffer A (50 mM sodium acetate, 5 mM DTT, pH 4.5) to 100% buffer B (50 mM sodium acetate, 500 mM NaCl, 5 mM DTT, pH 4.5) over 10 column volumes in 3 mL fractions. Ubiquitin started to elute at about 50% buffer B (around 250 mM NaCl). Fractions containing ubiquitin were pooled and concentrated with 4 mL, 3 kDa cutoff Amicon Ultra concentrators (Merck Millipore) to 6.4 mg/mL (MW = 8.6 kDa, ε_280nm_ = 1490 M^-1^cm^-1^) and flash-frozen in liquid nitrogen.

To covalently bind ubiquitin to the H3 peptide analogue, the G76C mutant of ubiquitin was buffer-exchanged in 50 mM sodium phosphate, pH 7.3, using a Zeba Spin desalting column, 7K MWCO, 2 ml according to the manufacturer’s protocols (Thermo Fisher Scientific) and mixed with a 20-fold molar excess of 5,5′-dithiobis-(2-nitrobenzoic acid) (DTNB, Sigma Aldrich). The mixture was incubated for 3 h at room temperature with end-over-end rotation and then buffer-exchanged into ligation buffer (20 mM Tris-HCl, pH 7.0, 50 mM NaCl and 1 mM EDTA) using a Zeba Spin desalting column, 7K MWCO, 2 ml (Thermo Fisher Scientific). The H3 peptide analogue was dissolved and reduced in 20 mM Tris-HCl, pH 7.5 buffer containing 5 mM DTT, and then buffer-exchanged into ligation buffer, and mixed with a 5-fold molar excess of activated ubiquitin G76C-DTNB for 1 h at room temperature. The reaction products were separated on a 1 ml Mono S 5/50 GL cation-exchange column (GE Healthcare) with a gradient from 0% buffer SA (20 mM Tris-HCl, pH 7.0, 50 mM NaCl, 1 mM EDTA) to 100% buffer SB (20 mM Tris-HCl, pH 7.0, 1000 mM NaCl, 1 mM EDTA) over 20 column volumes at 0.5 ml/min and a fraction size of 300 μl. Fractions were analyzed by SDS-PAGE. The majority of non- and mono-ubiquitinated H3 peptide analogue did not bind to the column. The majority of di-ubiquitinated H3 peptide analogue (H3Ub2-peptide) started to elute at around 50% buffer SB (about 525 mM NaCl) with a purity of >90% based on the SDS-PAGE gel. These fractions were pooled and concentrated with a 0.5 ml, 3 kDa cutoff Amicon Ultra concentrator (Merck Millipore) to 2.2 mg/ml (MW = 21.0 kDa, ε_280nm_ = 8730 M^-1^cm^-1^) while slowly exchanging the buffer towards storage buffer (25 mM HEPES pH 7.5, 50 mM NaCl) and flash-frozen in liquid nitrogen or used directly.

### Reconstitution of DNMT1: Non-productive DNA and DNMT1: Productive DNA complexes

To prepare the DNMT1: non-productive DNA complex in presence of inhibitor GSK3852279B, DNMT1 was mixed with the 26 base pair DNA duplex, S-adenosyl methionine (SAM, Merck) and GSK3852279B in 25 mM HEPES pH 7.5, 75 mM NaCl, 1 mM DTT. The protein: DNA: SAM: GSK3852279B ratio was 1:1.5:20:10 with 0.4 mg/ml DNMT1. The mixture was incubated for 30 min on ice and used for cryo-EM grid preparation.

To reconstitute the DNMT1: productive DNA complex in presence of the H3Ub2-peptide and to generate the covalent DNMT1:5FDNA complex, DNMT1 was first incubated with a 5-fold molar excess of H3Ub2-peptide in a buffer (25 mM Tris-HCl pH 7.5, 20 mM NaCl, 20% glycerol, 3 mM MgCl_2_) for 30 min on ice with a final DNMT1 concentration of 0.73 mg/ml. Then an 8-fold molar excess (to DNMT1) of the 26 base pair DNA duplex (5FDNA) was added and the mixture was further incubated on ice for 30 min. Finally, a 50-fold molar excess (to DNMT1) of SAM was added, incubated for 15 min on ice and the mixture was transferred to the shaking incubator set to 37°C for 1 h. The DNMT1: productive DNA complex was purified on a 0.8 ml Mini Q 4.6/50 PE column (GE Healthcare) with a gradient from 0% buffer QA (20 mM Tris-HCl, pH 7.5, 100 mM NaCl) to 100% buffer QB (20 mM Tris-HCl, pH 7.5, 1000 mM NaCl) over 20 column volumes at 0.5 ml/min and a fraction size of 50 μl. Fractions were subsequently analysed by a gradient 4–12% SDS-PAGE stained with SYBR™ Gold and Instant Blue for DNA and protein detection, respectively. The peak fraction corresponding to the DNMT1: productive DNA complex in presence of the H3Ub2-peptide was used directly for cryo-EM grid preparation.

In order to reconstitute the DNMT1 complex with H3Ub2-peptide alone, DNMT1 (4mg/ml final concentration) was incubated with a 1.5-fold molar excess of H3Ub2-peptide in a buffer (25 mM Tris-HCl pH 7.5, 50 mM KCl) for 1 h on ice. The sample was then directly used for cryo-EM grid preparation.

### Cryo-EM sample preparation and data collection

All grids were pre-treated in the plasma cleaner (Model 1070 NanoClean, 10% O_2_/90% Ar) for 45 sec. A total of 3.5 μl sample was applied on a grid under 100% humidity at 4°C and frozen in liquid ethane using the Vitrobot Mark IV System (Thermo Fisher Scientific).

Apo DNMT1 was frozen at 0.25 mg/ml using Quantifoil gold grids (Au 200 R1.2/1.3) and blotting conditions of waiting time 0 sec, blotting time 3 sec and blotting force 0. Images were taken at the calibrated magnification of 130,000x with the pixel size of 0.645 Å. Each micrograph was dose-fractionated to 40 frames with a total dose of about 40 e-/Å^2^. The defocus range was set from -0.5 μm to -1.6 μm.

The DNMT1: non-productive DNA complex in presence of inhibitor GSK3852279B was frozen at 0.4 mg/ml (DNMT1 concentration) using Quantifoil gold grids (Au 200 R1.2/1.3) and blotting conditions: wait time 0.5 sec, blot time 3 sec, blot force 0. Images were collected at the calibrated magnification of 130,000x with the pixel size of 0.645 Å. Each micrograph was fractionated into 40 frames with a total dose of about 40 e-/Å^2^. The defocus range was set from -0.5 μm to -1.6 μm.

The DNMT1: productive DNA complex in presence of the H3Ub2-peptide was frozen at 0.43 mg/ml (DNMT1 concentration) using Quantifoil gold grids (Au 200 R2/2) with blotting conditions: wait time 0 sec, blot time 0.5 sec, blot force 3. The images were collected at the calibrated magnification of 130,000x and the pixel size of 1.041 Å. Each micrograph was dose-fractionated to 26 frames with a total dose of about 40 e-/Å^2^. The defocus range was set from -0.7 μm to -2.0 μm.

DNMT1: H3Ub2-peptide at 4 mg/ml (DNMT1 concentration) was first mixed with β-octyl-glucoside (OG) to the final OG concentration of 0.3 CMC and applied on Quantifoil gold grids (Au 200 R2/2) and frozen. Blotting conditions were: wait time 0 sec, blot time 0 sec, blot force 3. Images were collected at the calibrated magnification of 105,000x and the pixel size of 1.327 Å. Each micrograph was dose-fractionated to 20 frames with a total dose of about 40 e-/Å2. The defocus range was set from -0.8 μm to -2.2 μm.

### Cryo-EM data processing

All micrographs were acquired on a Titan Krios microscope (Thermo Fisher Scientific) operated at 300 kV equipped with a K2 or K3 direct electron detector (Gatan) and recorded using SerialEM software. Depending on the dataset, a total of 3,500–5,300 micrographs were collected. Beam-induced motion was corrected by MotionCor2 [27]. The defocus parameters were estimated by CTFFIND4 [28]. Particles were picked using WARP [29]. The particles were then imported into cryoSPARC and the rest of the processing was done in cryoSPARC [30]. Each dataset was first subjected to 2D classification to clean the data and to remove ice. The well-featured 2D averages (230,000–450,000 particles) were used for *ab initio* reconstruction with 3–5 classes to generate the initial 3D model. The initial model was then used for 3D heterogeneous refinement with 3–10 classes. The best class was further refined using non-uniform refinement.

Available structures of DNMT1 (mentioned in the respective result sections) were fitted manually by rigid-body fitting in the electron density maps using UCSF ChimeraX v1.3. Figures were created using UCSF ChimeraX v1.3.

### EMSA and DNA methylation assay

DNMT1 binding to the 26 base pair non-covalent DNA duplex in the presence and absence of the GSK3852279B compound was analysed using electrophoretic mobility shift assay (EMSA). DNMT1 was mixed with a 2-fold molar excess of DNA and a 20-fold molar excess of SAM in binding buffer (25 mM Tris-HCl, pH 7.5, 40 mM NaCl, 20% glycerol, 1 mM DTT, 0.5 mM MgCl_2_). The mixture was then split in two parts, one part was supplemented with a 5-fold molar excess of GSK3852279B, the second control part was supplemented with binding buffer. Reactions were incubated overnight at room temperature and analysed with 4% native Tris-glycine PAGE stained with SYBR™ Gold and Instant Blue.

Subsequently, we assessed the ability of DNMT1 to form a covalent complex with the 26 base pair DNA duplex carrying 5-fluorodeoxycytosine (5FDNA), which can only occur after enzyme activity. In order to activate the protein, DNMT1 was pre-incubated with a 10-fold molar excess of H3Ub2-peptide for 15 min on ice in reaction buffer (25 mM Tris-HCl, pH 7.5, 20 mM KCl, 20% glycerol and 2 mM MgCl_2_). The mixture was then supplemented with a 5-fold molar excess (to DNMT1) of 5FDNA, incubated for 30 min on ice and split in two parts. One was supplemented with a 2.5-fold molar excess of GSK3852279B; the other was supplemented with the reaction buffer as negative control. The reactions were started by addition of SAM (150-fold excess) and incubated at 37°C for 1 h. The reaction products were supplemented with a reducing SDS-PAGE loading buffer, boiled for 5 min at 95°C to disrupt non-covalent interactions and separated on a gradient 4–12% SDS-PAGE, subsequently stained with SYBR™ Gold and Instant Blue.

### LC-MS/MS mass spectrometry of full length DNMT1

To verify the completeness of full length DNMT1 after protein purification, mass spectrometry was performed. Coomassie-stained bands were excised, chopped into small pieces and transferred to 0.5 ml Eppendorf tubes. For all following steps, buffers were exchanged by two consecutive 15 min incubation steps of the gel pieces with 200 μl of acetonitrile (ACN) whereby ACN was removed after each step. Proteins were reduced by the addition of 200 μl of a 10 mM Dithiothreitol (DTT) solution in 100 mM ammonium bicarbonate (AmBiC). Samples were incubated at 56°C for 20 min, 180 μl ACN were added and samples were incubated for 15 min incubation at room temperature. Proteins were alkylated for 20 min by the addition of 200 μl of a 55 mM chloroacetamide (CAA) solution in 100 mM AmBiC. Gel pieces were incubated twice with 200 μl ACN for 15 min at room temperature. Next, proteins were digested by acid hydrolysis. Dried gel pieces were transferred to glass vials (Chromacol glass inserts; Thermo Scientific) and placed into a 2 ml Eppendorf cup with 700 μl of H_2_O. 50 μl of 3 M HCl were added and gel pieces were incubated for 5 min at room temperature. Samples were then transferred into a microwave where they were heated for 10 min at 1.000 W. Samples were spun down and the supernatant was directly subjected to a reverse phase clean-up step (OASIS). Peptides were dried in a speedvac and reconstituted in 10 μl of an aqueous solution of 0.1% (v/v) formic acid. Peptides were analyzed by LC-MS/MS on an Orbitrap Fusion Lumos mass spectrometer (Thermo Scientific) as previously described [31]. To this end, peptides were separated using an Ultimate 3000 nano RSLC system (Dionex) equipped with a trapping cartridge (Precolumn C18 PepMap100, 5 mm, 300 μm i.d., 5 μm, 100 Å) and an analytical column (Acclaim PepMap 100. 75 × 50 cm C18, 3 mm, 100 Å) connected to a nanospray-Flex ion source. The peptides were loaded onto the trap column at 30 μl per min using solvent A (0.1% formic acid) and peptides were eluted using a gradient from 2 to 85% Solvent B (0.1% formic acid in acetonitrile) over 30 min at 0.3 μl per min. The Orbitrap Fusion Lumos was operated in positive ion mode with a spray voltage of 2.2 kV and capillary temperature of 275°C. Full scan MS spectra with a mass range of 375–1200 m/z were acquired in profile mode using a resolution of 120,000 (maximum injections time of 50 ms, AGC target was set to 400% and a max injection time of 86 ms. Precursors were isolated using the quadrupole with a window of 1.2 m/z and Fragmentation was triggered by HCD in fixed collision energy mode with fixed collision energy of 34%. MS2 spectra were acquired with the Orbitrap with a resolution of 30.000 and a max injection time of 86 ms. Acquired data were analyzed using IsobarQuant [32] and Mascot V2.4 (Matrix Science) using a reverse in house generated FASTA database from Sf21 (*Spodoptera frugiperda*) including common contaminants. The following modifications were taken into account: Carbamidomethyl (C, fixed), Acetyl (K, variable), Acetyl (Protein N-term, variable) and Oxidation (M, variable). The mass error tolerance for full scan MS spectra was set to 10 ppm and to 0.02 Da for MS/MS spectra. A maximum of 2 missed cleavages were allowed. A minimum of 2 unique peptides with a peptide length of at least seven amino acids and a false discovery rate below 0.01 were required on the peptide and protein level [33].

## Results

### The 3.3 Å cryo-EM structure of apo human DNMT1

DNMT1 is a multi-domain protein comprising a regulatory N-terminal and a catalytic C-terminal region (Fig 1A). Previous structural studies on DNMT1 provided solid evidence for DNMT1’s high dynamics and its need for conformational rearrangements to perform the catalytic reaction [9, 14, 24, 34]. These studies were conducted on truncated DNMT1 that included a minimal enzymatically active fragment and therefore missed the first N-terminal ~350 amino acids. In our study, we aimed to analyse full-length DNMT1 to address DNMT1’s conformational flexibility in the context of the entire molecule. Considering DNMT1’s dynamic nature, we set out to analyse DNMT1 by single-particle cryo-EM that is especially advantageous for the analysis of dynamic and heterogeneous assemblies. Cryo-EM preserves proteins at a close-to-native state and enables the sorting of discrete conformational and compositional states [35]. In addition, cryo-EM rapidly has become the method of choice in structure-based drug design for proteins [36] that are challenging to study by X-ray crystallography or NMR.

As the first step, we analysed full-length apo human DNMT1 to pinpoint the molecule’s most flexible regions and correlate them with DNMT1’s functionality. We confirmed the correct size and mass of full-length DNMT1 by SDS-PAGE and mass spectrometry (S1 Fig). To obtain optimal particle orientation and distribution on the cryo-EM grid, we tested different DNMT1 concentrations, several blotting conditions and different support grids. The optimized sample was then subjected to cryo-EM imaging (Fig 1). Analysis of the 2D class averages suggested an overall good quality of the collected dataset, showing monomeric DNMT1 with well-defined structural features (Fig 1B). Subsequent *ab initio* 3D reconstruction and classification resulted in a map with an overall estimate of 3.3 Å resolution that extends even further, up to 2.9 Å, in the core of the protein (Table 1, Fig 1C and 1D). The estimated resolution is in a good agreement with the level of molecular details seen within this map. It allowed unambiguously distinguishing of the secondary structure elements and placement of side chains, not only large bulky residues but also those of smaller size (Fig 1E).

**Table 1:** Cryo-EM data collection of apo DNMT1, DNMT1: Non-productive DNA complex, DNMT1: H3Ub2-peptide, DNMT1: Productive DNA complex with refinement statistics for apo DNMT1.

	apo DNMT1	DNMT1 non-productive DNA complex in presence of inhibitor GSK3852279B	DNMT1: H3Ub2 DNMT1 bound to the H3Ub2-peptide	DNMT1 productive DNA complex in presence of the H3Ub2-peptide
**EMDB identifier**	EMD-18418	EMD-50795	EMD-50801	EMD-50802
**Data collection**				
Magnification	130,000	130,000	105,000	130,000
Voltage (kV)	300	300	300	300
Electron exposure (e–/Å^2^)	40	40	40	40
Defocus range (μm)	0.5–1.6	0.5–1.6	0.8–2.2	0.7–2.0
Pixel size (Å)	0.645	0.645	1.327	1.041
Number of images	4320	2804	5351	2926
Initial particle images (no.)	507,910	456,731	741,000	306,475
Final particle images (no.)	140,304	20,006	81,568	133,447
Map resolution (Å)	3.3	5.1	6.0	4.6
FSC threshold	0.143	0.143	0.143	0.143
Map resolution range (Å)	2.9–6.7	4.9–10.0	5.5–11.1	4.3–10.5
**Refinement**				
Initial model used	PDB ID 4WXX			
Model resolution (Å)	3.5			
FSC threshold	0.5			
Map sharpening *B* factor (Å^2^)	N/A			
**Model composition**				
Non-hydrogen atoms	9501			
Protein residues	1171			
Water	311			
Ligands	5x Zn			
***B* factors (Å** ^**2**^ **)**				
Protein (min/max/mean)	15.5/150.8/58.4			
Water (min/max/mean)	12.9/98.2/42.3			
Ligand (min/max/mean)	79.3/167.5/121.9			
**R.m.s. deviations**				
Bond lengths (Å) (#>4σ)	0.014 (0)			
Bond angles (°) (#>4σ)	1.411 (14)			
**Validation**				
MolProbity score	1.77			
Clashscore	5.26			
Rotamer outliers (%)	0.21			
**Ramachandran plot**				
Favored (%)	91.93			
Allowed (%)	7.90			
Disallowed (%)	0.17			
**Rama-Z score**				
Whole (N = 1152)	-3.49 (0.20)			
Helix (N = 282)	-3.10 (0.20)			
Sheet (N = 148)	-1.34 (0.39)			
Loop (N = 722)	-2.29 (0.20)			

The overall architecture of apo DNMT1 obtained from the cryo-EM reconstruction closely resembles the crystal structures of apo mouse and human DNMT1 [14, 15] and includes RFTS, CXXC, autoinhibitory linker (AL), BAH1, BAH2 and MTase domains (Fig 1A and 1C). To explain the cryo-EM map, an atomic model of apo DNMT1 was placed by rigid-body fitting using the crystal structure of human DNMT1 (PDB ID 4WXX) [14]. The DNMT1 cryo-EM map is also in good agreement with the recently reported cryo-EM reconstruction of truncated human DNMT1 (residues 351–1616) [24]. Notably, although full-length DNMT1 was used for our cryo-EM analysis, the part of the N-terminus that precedes the RFTS domain is not visible in the cryo-EM reconstruction (Fig 1C). This part is also lacking in the crystal structures of apo human and mouse DNMT1 [14, 15], where it was removed in order to obtain crystals.

The cryo-EM map corresponding to the MTase domain shows the highest resolution of 2.9 Å, owing to its core position within the protein, while RFTS and CXXC domains are resolved to a lesser extent (Fig 1D). The lowest resolution is observed in the CXXC domain and its connecting region to the RTFS domain, the AL and the loop of BAH2 that are also partly disordered in the crystal structure. A similar resolution distribution is also observed in the cryo-EM reconstruction of the truncated human DNMT1 [24]. Consistent with these observations, these DNMT1 regions are suggested to undergo conformational rearrangements in order to assist the transition of DNMT1 between different functional states.

### Non-productive DNMT1-DNA complex in the presence of a DNMT1-selective inhibitor

DNMT1 is a promising target for anticancer therapy that aims to regulate the DNA methylation activity of DNMT1 by small molecules [37]. Previously published crystal structures of truncated DNMT1 in complex with DNA provided significant insights into the understanding of the molecular mechanism of DNA methylation by DNMT1 and its possible regulation modes [9, 34]. They show that unmethylated DNA is excluded from the active site of DNMT1, preventing its methylation, while a hemi-methylated DNA can form a productive complex and becomes efficiently methylated.

Recent crystallographic studies of truncated DNMT1 with DNA and a selective inhibitor show how the compound competes with the active-site loop of DNMT1 for penetration into hemi-methylated DNA and prevents DNA methylation [6]. Another study performed the structural and functional characterization of different DNMT1 inhibitors bound to the DMT1: DNA complex, including the GSK3852279B compound used by us (PDB ID 7SFF, [25]) providing additional insights into the competition with the active site loop. Using cryo-EM, we now addressed the DNA binding activity of DNMT1 in the presence of the small molecule DNMT1 inhibitor, GSK3852279B, in the context of the full-length protein. Compared to the previous structural studies using x-ray crystallography, we aimed to determine the structure of the non-productive complex with full-length DNMT1 to get a better mechanistic understanding.

**Fig 2:**
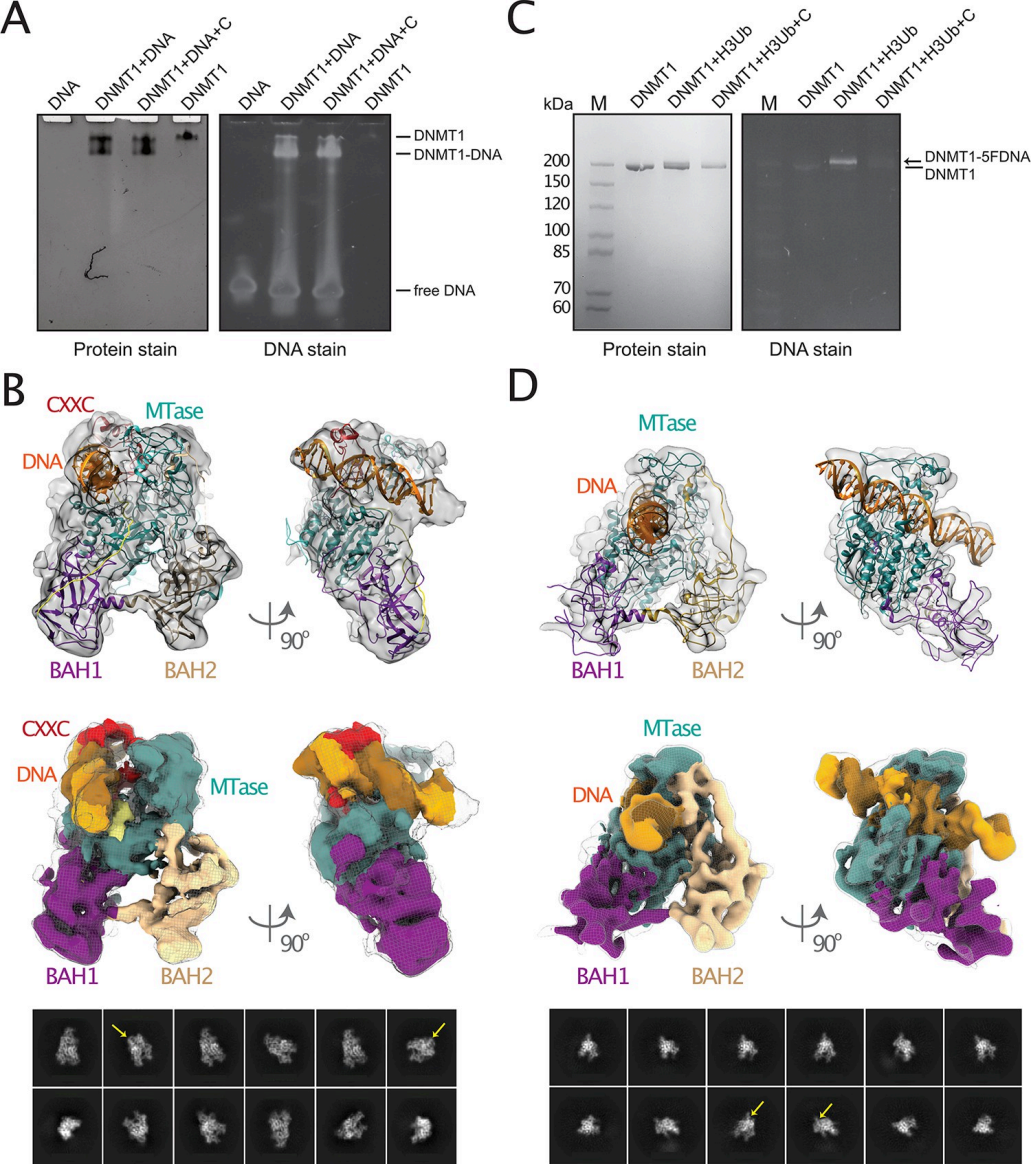
DNMT1 interaction with hemi-methylated DNA. (A) Binding of DNMT1 to DNA in the presence and absence of GSK3852279B inhibitor (labeled as [C]) analysed by native gel electrophoresis. The gel is stained to detect either protein (left) or DNA (right). (B) Cryo-EM density map and 2D analysis of the DNMT1: DNA: GSK3852279B complex showing the non-productive state. The DNMT1 domains are colored as in Fig 1A. The DNA density is indicated by yellow arrows (2D) and colored in orange (3D). Crystal structure PDB 3PTA [34] has been placed into the cryo-EM density. (C) DNA methylation by DNMT1 in the presence of H3Ub2-peptide and GSK3852279B inhibitor (labeled as [C]) analyzed by SDS-PAGE. The gel is stained to detect either protein (left) or DNA (right). Free DNMT1 and DNMT1 cross-linked to 5FDNA (DNMT1-5FDNA) are indicated. (D) Cryo-EM density map and 2D analysis of the DNMT1:5FDNA complex in the presence of H3Ub2-peptide showing the productive state. The DNMT1 domains are colored as in Fig 1A. The DNA density is indicated by yellow arrows (2D) and colored in orange (3D). Crystal structure PDB 4DA4 [9] has been placed into the cryo-EM density.

We first analysed binding of full-length DNMT1 to a 26-base pair (bp) hemi-methylated DNA with a single hemi-mCpG site (Fig 2A, S2A and S2B Fig). The double stranded DNA contains a central hemi-methylated CpG site (mC) for proper substrate recognition. Previous results showed that the RFTS domain acts as a competitor of DNA for the binding to the catalytic MTase domain resulting in weak DNA binding [16, 38]. We then compared DNA binding in the presence of the GSK3852279B compound. Previous crystallographic data of a similar compound suggest that the compound intercalates between DNA bases and does not interfere with DNA binding by DNMT1 [6]. In addition, similar inhibitors could interact with the active-site loop while intercalated in the target DNA [25]. In agreement, we did not observe a substantial change in DNA binding of DNMT1 in the presence of the compound (Fig 2A, S2A and S2B Fig).

We next reconstituted the DNMT1: DNA complex in the presence of GSK3852279B and analysed it using cryo-EM (Fig 2B). Following multiple rounds of sample optimization (varying concentration, buffers, blot parameters, and testing different grids), we collected cryo-EM data followed by their analysis (Fig 2B). The 2D classification indicated compositional heterogeneity of the sample and suggested the presence of two major particle species. One species corresponded to apo DNMT1, while the second one displayed additional features indicating the presence of DNA (Fig 2B, bottom panel: yellow arrows). Subsequent 3D analysis confirmed that only a small fraction of the DNMT1 particles was bound to DNA (Fig 2B). To interpret the obtained cryo-EM density map, we fitted the available crystal structures of the DNA-bound DNMT1 that represent either the non-productive auto-inhibited DNA-binding [34] (PDB ID 3PTA) or the productive DNA-binding conformation [9] (PDB ID 4DA4, mouse DNMT1). Interestingly, although we used hemi-methylated DNA to assemble the complex and could have expected the formation of the productive DNMT1: DNA complex, our cryo-EM reconstruction closely resembles the structure of DNMT1 in complex with unmethylated DNA [34] (PDB ID 3PTA). Consistently, the functional domains CXXC, AL, BAH1, BAH2 and MTase could be placed in the density. In contrast, no cryo-EM density for the compound itself, which is not surprising given that the compound only binds to a productive DNMT1: DNA complex. Notably, the autoinhibitory RFTS domain is not visible in the cryo-EM map (Fig 2B). This suggests the flexibility of the RFTS domain and indicates that it needs to undergo structural rearrangements in order to accommodate the DNA. The different crystal structures of human and mouse DNMT1 (PDB identifiers 3PTA, 4DA4, 7SFF) were all solved with truncated DNMT1 lacking the flexible N-terminus including the RFTS domain [9, 25, 34]. As our structure compares well with these structures, and mass-spectrometry results confirm the presence of full-length DNMT1, we conclude that compared to our apo DNMT1 structure the regulatory RFTS domain was displaced by DNA binding and stays flexible without inducing major conformational changes to the other domains. In conclusion, our cryo-EM reconstruction of DNMT1 with DNA and inhibitor GSK3852279B shows that the hemi-methylated DNA is bound in a non-productive state and, thereby, excluded from the active site preventing its methylation. Interestingly, crystal structures with similar inhibitors and hemi-methylated DNA showed a productive conformation of DNMT1 [25]. However, the truncated DNMT1 proteins used in these studies are lacking the RFTS domain which could still–albeit disordered in our cryo-EM structure–influence the DNA binding. This could especially be the case without ubiquitinated H3 histone which activates and interacts with the RFTS domain.

### Ubiquitinated H3 histone promotes formation of the productive DNMT1: DNA complex

The DNMT1 activity *in vivo* is subjected to tight regulation that allows proper positioning of DNMT1 on hemi-methylated DNA [39]. Among other factors, di-ubiquitinated histone H3 enhances the activity of DNMT1 towards hemi-methylated DNA and supports its correct cellular localization [22, 23, 40]. Recently, Kikuchi et al. published cryo-EM maps and structures of apo DNMT1, DNMT1: di-ubiquitinated-H3-peptide and two DNMT1: di-ubiquitinated-H3-peptide: hemi-methylated DNA complexes with different CXXC conformations (PDB IDs 7XI9, 7XIB) using an N-terminally truncated DNMT1 (residues 351–1616) [24]. The structures highlight rearrangements of the CXXC, autoinhibitory linker, and RFTS domains upon activation. We, therefore, aimed to better understand the mechanism of DNMT1 activation by di-ubiquitinated histone H3 using full-length DNMT1 to complete the picture.

We first prepared the histone H3 peptide analogue, dual mono-ubiquitylated at position K18 and K23 (H3Ub2-peptide), previously shown to be an effective activator of DNMT1 [22]. As described in detail in the method section, the H3 peptide analogue carried K18C and K23C mutations and was linked by disulfide bonds with two G76C-mutated ubiquitin molecules. H3Ub2-peptide was purified from non- and mono-ubiquitinated H3 peptide analogues with >90% purity (S3 Fig). To demonstrate that DNMT1 was indeed activated by the di-ubiquitinated H3Ub2-peptide, and can bind DNA in the catalytically competent conformation, followed by DNA methylation, we used a double-stranded DNA that contained 5-fC on the target DNA strand (5FDNA). The 5-fC-containing DNA is an inhibitor of DNA methyltransferases that leads to the formation of an irreversible, covalent complex with DNMT1 after the enzymatic reaction [9, 41]. The sequence and length of the 5FDNA duplex were otherwise identical to the DNA duplex used to reconstitute the DNMT1: DNA: GSK3852279B complex in Fig 2B. Although we did not test the catalytic activity of DNMT1, only when the H3Ub2-peptide was added to the reaction the formation of the covalent, irreversible DNMT1:5FDNA complex could be observed (Fig 2C, S2C and S2D Fig).

Once we biochemically confirmed the formation of the DNMT1:5FDNA complex in the presence of the H3Ub2-peptide, we subjected it to cryo-EM analysis (Fig 2D). To separate the non-reacted, free DNMT1 from the DNMT1:5FDNA: H3Ub2-peptide complex, we performed anion exchange chromatography and collected the corresponding fractions containing the complex (S4 Fig). We then prepared cryo-EM grids, collected and processed the data. The 2D classification showed clear density for the DNMT1-bound DNA molecule (Fig 2D, bottom panel: yellow arrows). The subsequent 3D reconstruction allowed unambiguous placement of DNMT1 and DNA. Consistent with our biochemical data, the cryo-EM map agrees very well with the published crystal structure of the productive covalent DNMT1: DNA complex [9] (PDB ID 4DA4) and includes BAH1, BAH2, and Mtase domains (Table 1).

Recently, Kikuchi et al. also published the cryo-EM structure of the truncated human DNMT1 (residues 351–1616) in complex with 14-base pair 5FDNA and H3Ub2-peptide [24]. In this structure, one subset of the particles included ordered CXXC, while in another subset of the particles CXXC is disordered. In our cryo-EM reconstruction, we observe an additional weaker density in the putative CXXC region. However, the confident placement of CXXC in the correct orientation is limited by the low resolution of the cryo-EM map. Therefore, it might be that our reconstruction represents the average of the two types of particles described by Kikuchi et al..

Like in our cryo-EM reconstruction of the non-productive DNMT1: DNA complex, we did not observe any density for the autoinhibitory RFTS domain. The density for the 5FDNA, however, covered the entire length of 26 base pairs suggesting that DNA is bound more stably and rigid in the productive complex (Fig 2D) which closely resembles the crystal structure of human DNMT1 in complex with hemi-methylated DNA and the GSK3852279 inhibitor (PDB ID 7SFF, [25]) compared to the non-productive complex (Fig 2B). Interestingly, although H3Ub2 peptide was present in the sample, we did not find DNMT1 complexes with cryo-EM density corresponding to the H3Ub2-peptide. This is consistent with the above-mentioned cryo-EM structure of Kikuchi et al. that also showed no density for both RFTS and H3Ub2.

### Cryo-EM map of apo human DNMT1 bound to di-ubiquitinated histone H3 peptide

We next directed our efforts towards providing a more comprehensive understanding of the structural basis of DNMT1 activation by H3Ub2. The molecular mechanism of this activation is described mainly based on the available crystal structures of the isolated RFTS domain in complex with either two ubiquitin molecules (PDB ID 5YDR), dual mono-ubiquitylated H3 (H3Ub2) (PDB ID 5WVO) or with H3K9me3/H3Ub histone (PDB ID 6PZV) [22, 23, 42]. These results suggested that binding of these components to the RFTS domain causes a bend in the helix α4 of RFTS, which weakens the interactions of the RFTS with the Mtase domain and opens the catalytic pocket. The cryo-EM structure of N-terminally truncated DNMT1 (aa 351–1616) with H3Ub2 suggested that H3Ub2 binding alone does not lead to the displacement of the RFTS domain from the catalytic core [24] (PDB IDs 7XI9, 7XIB). However, part of the RFTS domain and the bound H3Ub2-peptide were not visible in their reconstruction and a truncated version of DNMT1 was used [24]. To understand H3Ub2 binding to full-length DNMT1, we analysed the DNMT1: H3Ub2-peptide complex by cryo-EM.

**Fig 3:**
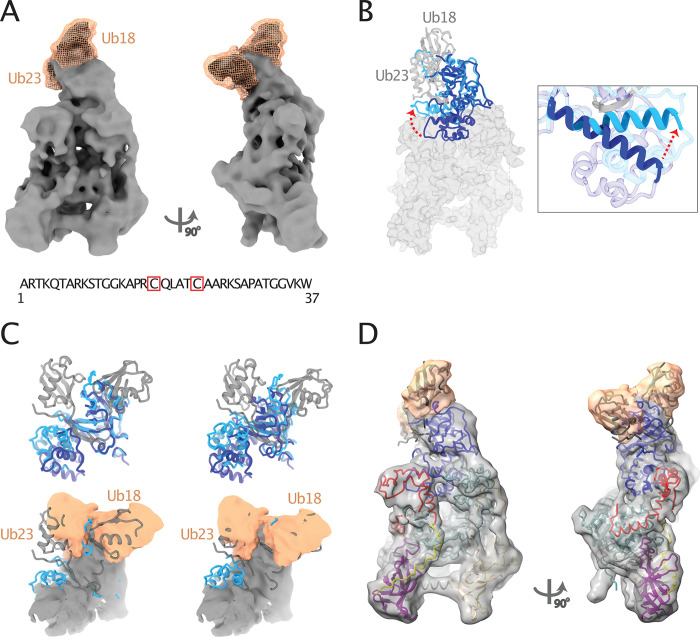
Cryo-EM map of full-length DNMT1 in complex with a di-ubiquitinated histone H3 peptide (H3Ub2-peptide). (A) Cryo-EM density map showing DNMT1 (grey) and additional densities for the two ubiquitin molecules of the histone H3 peptide analogue (wheat) in front and side views. The sequence of the H3 peptide and the positions of Ub-18 and Ub-23 attached to it (red boxes) are indicated in the lower panel. (B) Superposition of the RFTS domain from apo DNMT1 (PDB ID 4WXX; RFTS in dark blue; other DNMT1 domains shown in grey surface) and RFTS from the RFTS-H3Ub2 crystal structure (PDB ID 5WVO; RFTS in light blue; ubiquitin molecules in grey cartoon). The bend in the α4-helix of RFTS is indicated by a red arrow and shown in the close-up view on the right. (C) Two alternative fits (on the left and right) of the RFTS: H3Ub2 crystal structure (PDB ID 5WVO, light blue RFTS, grey H3Ub2) into the cryo-EM density map of DNMT1: H3Ub2-peptide (lower panel). RFTS domain from the apo DNMT1 crystal structure (dark blue, upper panel) is provided as reference. (D) Fit of the apo DNMT1 structure (PDB ID 4WXX, domains colored as in Fig 1A) and the two ubiquitin molecules from the RFTS: H3Ub2 structure (PDB ID 5WVO) into the cryo-EM map of DNMT1: H3Ub2-peptide.

We assembled full-length DNMT1 in complex with the H3Ub2-peptide and prepared the sample for cryo-EM analysis. Initial trials to prepare a sample suitable for cryo-EM analysis were unsuccessful. We therefore decided to add mild detergent to our sample prior to freezing, in order to prevent possible complex dissociation at the air-water interface and to minimize the preferred orientation of particles. This procedure led to a 3D reconstruction that contained apo DNMT1 and showed additional density adjacent to the RFTS domain (Table 1, Fig 3A). The additional density closely resembled ubiquitin molecules with the expected size and shape. We therefore attempted to fit the available crystal structure of the RFTS: H3Ub complex [22] (PDB ID 5WVO) into the cryo-EM reconstruction to account for the additional density and to assess the bend in helix α4 of the RFTS domain (Fig 3B and 3C). The RFTS domain and both ubiquitin molecules could not be fitted completely into the cryo-EM density assuming the same conformation as in the crystal structure (Fig 3C). The ~35 degree bend of helix α4 in the RFTS domain crystal structure causes part of the RFTS domain sticking out of the density, when both ubiquitin molecules are fitted in the respective cryo-EM density (Fig 3C, lower panel, right). Vice versa, when the RFTS domain is accommodated by the corresponding cryo-EM density, the two ubiquitin molecules do not align with the cryo-EM map (Fig 3C, lower panel, left). When the conformation of the RFTS domain was retained as observed in apo DNMT1, both, RFTS and two ubiquitin molecules, could be placed into the cryo-EM density (Fig 3D). This suggests that the bend of helix α4, observed in the crystal structure of the isolated RFTS domain bound to H3Ub2 (PDB ID 5WVO), might be less prominent in the context of full-length DNMT1. Thus, our cryo-EM reconstruction of the DNMT1: H3Ub2-peptide complex shows for the first time how H3Ub2 binds to full-length DNMT1. Consistent with Kukichi et al. [24], our results suggest that the binding of H3Ub2 alone does not lead to substantial conformational rearrangements of the RFTS domain and that the RFTS domain remains associated with the catalytic domain.

## Discussion

**Fig 4:**
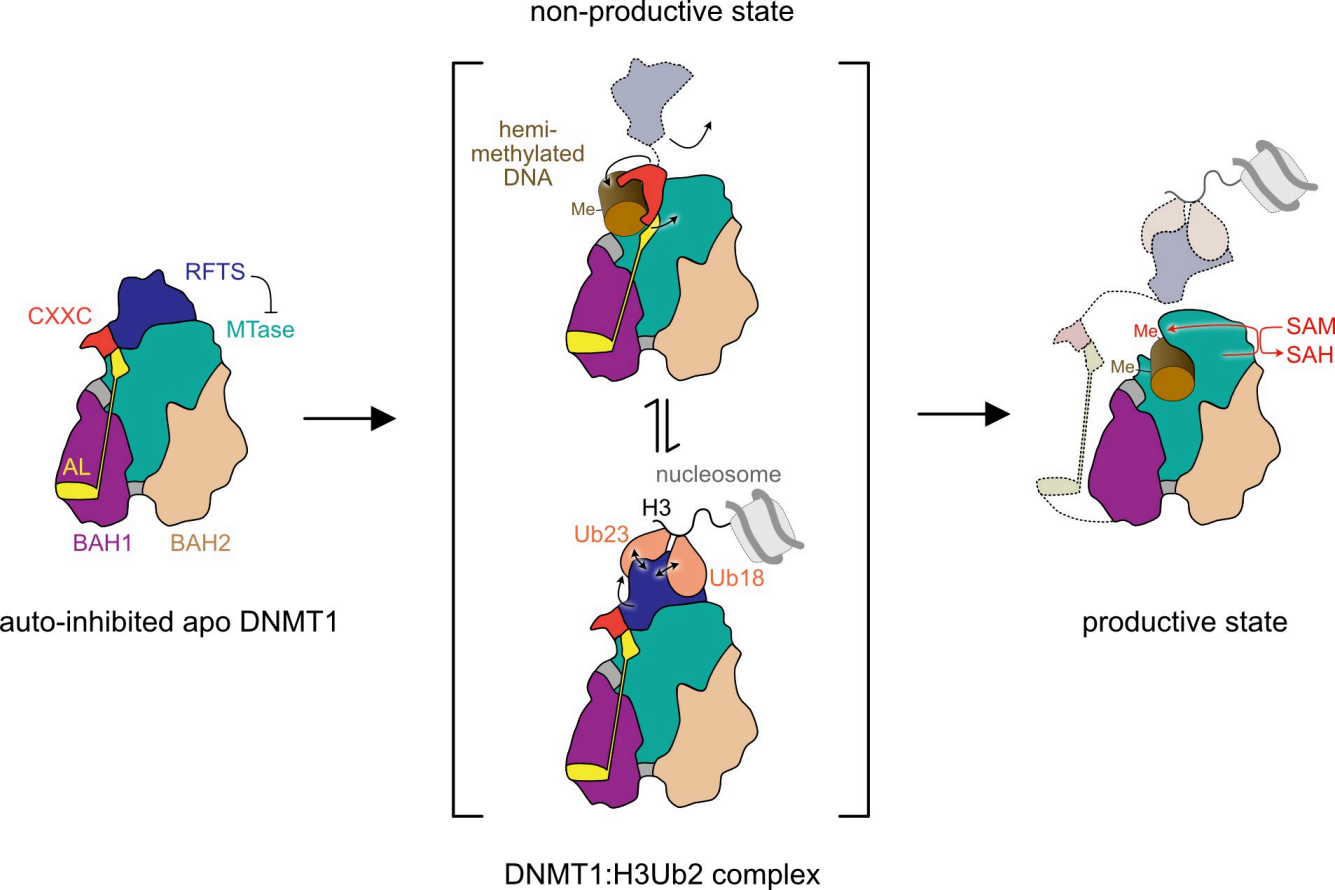
Schematic diagram of the DNMT1 reaction cycle. Apo DNMT1 is in an auto-inhibited state with the RFTS domain blocking access of the DNA to the catalytic domain (left panel). Binding of hemi-methylated DNA in a non-productive state and/or di-ubiquitinated histone H3 releases the autoinhibition of apo DNMT1 (middle panel). DNMT1 transitions into a productive state where hemi-methylated DNA can enter the active site to become fully methylated (right panel).

DNMT1-mediated DNA methylation is tightly controlled in space and time and requires (i) high degree of protein flexibility allowing DNMT1’s transition from the non-productive to the productive state and (ii) involvement of external protein binding partners that ensure DNMT1’s correct positioning on the DNA target site. The dynamic nature of DNMT1’s structural rearrangements plays a central role in its ability to accommodate hemi-methylated DNA and to induce the substrate-specific geometry for subsequent DNA methylation (Fig 4). In agreement, our cryo-EM maps show high flexibility of DNMT1 domains even resulting in the lack of density for some of them. Thus, in the apo form, high-resolution cryo-EM density for the nearly complete DNMT1 indicates a rather stable overall conformation (Figs 1C and 4, left panel). However, apo DNMT1 adopts an auto-inhibited conformation as the RFTS domain blocks the catalytic pocket and the RFTS domain has to be displaced to allow access to the DNA binding site [14].

In the cryo-EM structure of the non-productive DNMT1: DNA complex obtained in the presence of a DNMT1-selective inhibitor the RFTS domain is no longer visible (Figs 2B and 4, middle panel, top). The observed DNA binding mode of hemi-methylated DNA resembles the one observed for unmethylated DNA [34]. Recent crystal structures of the truncated human DNMT1 (residues 729–1600) in complex with chemically similar compounds suggest a possible mode of action as it shows a productive DNMT1-DNA complex (prior to the methylation reaction), where the compounds intercalate between the two C: G base pairs of DNA and prevent access of the DNMT1 active site loop to the target cytosine base [6, 25]. Molecular dynamics simulations of DNMT1: DNA interactions with modified DNA containing an oxidized form of mC [43] demonstrated that DNMT1 forms a non-productive complex because of altered interactions of the Mtase domain with the C5 substituent of cytosine. In accordance, it is possible that GSK3852279B can alter the target cytosine’s chemistry and prevents insertion of the active site loop thereby triggering conformational changes in DNMT1 that displace DNA from the active site resulting in a non-productive complex.

Di-ubiquitylated histone H3 of nucleosomes is an important signal for the recruitment of DNMT1 to the sites of DNA replication and for the subsequent enhancement of the DNMT1 methyltransferase activity [22]. Previous crystal structures and molecular dynamics stimulation visualized H3Ub2 interaction with the isolated RFTS domain and suggested gross conformational changes in the structure of the entire DNMT1 molecule in order to open the active site [22, 39]. We obtained an experimental cryo-EM map (although limited in resolution) that shows the interaction between the H3Ub2-peptide and DNMT1 in the context of the full-length protein (Figs 3 and 4, middle panel, bottom). The observed H3Ub2-peptide binding to the RFTS domain in full-length DNMT1 is similar to the H3Ub2 binding to the isolated RFTS domain [14]. However, the overall structure of DNMT1 in the DNMT1: H3Ub2-peptide complex does not support gross conformational rearrangements and, instead, closely resembles the apo DNMT1 structure (Fig 3C and 3D), in which the autoinhibiting RFTS domain maintains most interaction with the catalytic domain. Consistently, recent cryo-EM and SAXS analysis of DNMT1: H3Ub2 complex with an N-terminally truncated DNMT1 (residues 351–1616) by Kikuchi et al. reached similar conclusions and suggested that H3Ub2 binding does not completely displace the RFTS domain from the catalytic core [24]. However, the structural rearrangements between RFTS and catalytic domain in the DNMT1: H3Ub2-peptide complex might be sufficient for the hemi-methylated DNA to penetrate the active centre. Therefore, it is possible that we trapped an initial DNMT1: H3Ub2-peptide complex that did not yet become activated. Alternatively, the conformational changes of DNMT1 are less prominent than initially postulated and local changes in RFTS interactions with the catalytic domain are sufficient to trigger active site opening.

Finally, the transformation to the productive catalytic state of DNMT1 leads to further domain rearrangements and, in addition to the RFTS domain, also displaces the CXXC and AL regions allowing DNA binding in the productive conformation (Fig 4, right panel and [24]). Taken together, our results suggest that flexibility of DNMT1 increases as the enzyme transits through the catalytic cycle and that major conformational rearrangements accompany DNA binding and subsequent DNA methylation. In this manuscript, we are adding our structural insights resulting from the cryo-EM analysis using full-length DNMT1 to the already available structural information (S1 Table). In addition, although we used full-length DNMT1, the entire N-terminal region preceding the RFTS is not visible in any of our cryo-EM maps reflecting the high flexibility of this region. This region includes the PIP box that interacts with PCNA to tether DNMT1 to the replication fork. Future studies may address the contribution of DNMT1’s N-terminal region in the enzyme’s structural dynamics.

## Supporting information

S1 Fig Mass spectrometry of full-length DNMT1. (A) Coomassie-stained SDS-PAGE gel of purified full-length DNMT1 at varying concentrations. The red boxes mark samples analyzed by mass spectrometry. (B) Mass spectrometry result using acid hydrolysis confirms the presence of full-length (N-terminally tagged) DNMT1 as indicated by the identified peptides over the full range of DNMT1.
(TIF)

S2 Fig Full SDS-PAGE gel images of the DNMT1 binding assays. (A-B) Binding of DNMT1 to DNA in the presence and absence of GSK3852279B inhibitor (labeled as [C]) is analysed by native gel electrophoresis. The gel is stained to detect either protein (A) or DNA (B). (C-D) DNA methylation by DNMT1 in the presence of H3Ub2-peptide and GSK3852279B inhibitor (labeled as [C]) is analyzed by SDS-PAGE. The gel is stained to detect either protein (C) or DNA (D). Free DNMT1 and DNMT1 cross-linked to 5FDNA (DNMT1-5FDNA) are indicated. Boxes indicate the cropped area used in Fig 2.
(TIF)

S3 Fig Purification of di-ubiquitinated H3 analogue H3Ub2-peptide. (A) H3Ub2-peptide was purified on a Mono S 5/50 GL chromatography column using a salt concentration gradient (concentration buffer B). (B) Fractions were analyzed by SDS-PAGE. The arrow and bold fraction numbers indicate the main peak of H3Ub2-peptide with >90% purity over mono-ubiquitinated (H3Ub1) or non-ubiquitinated (H3) species.
(TIF)

S4 Fig Purification of the DNTM1: Productive DNA complexes. (A) The DNMT1:5FDNA: H3Ub2-peptide complex was purified using a MiniQ 4.6/50 PE chromatography column using a salt concentration gradient (concentration buffer B). Fractions were analyzed by SDS-PAGE using a protein stain (B) and a DNA stain (C). The arrow and bold fraction number indicate the main peak of the productive complex.
(TIF)

S1 Table Overview of published DNMT1 structures. afull length DNMT1 was used. (DOCX)

## Abbreviations

DNMT: DNA methyltransferase
cryo-EM: cryo-electron microscopy
Ub: ubiquitin
DTT: dithiothreitol
H3: histone H3
