# Supplementary figures and images for: Structural insight into the DNMT1 reaction cycle by cryo-electron microscopy
Source: PLoS One. 2024 Sep 3;19(9):e0307850. doi: 10.1371/journal.pone.0307850 (PMC11371216; doi:10.1371/journal.pone.0307850)

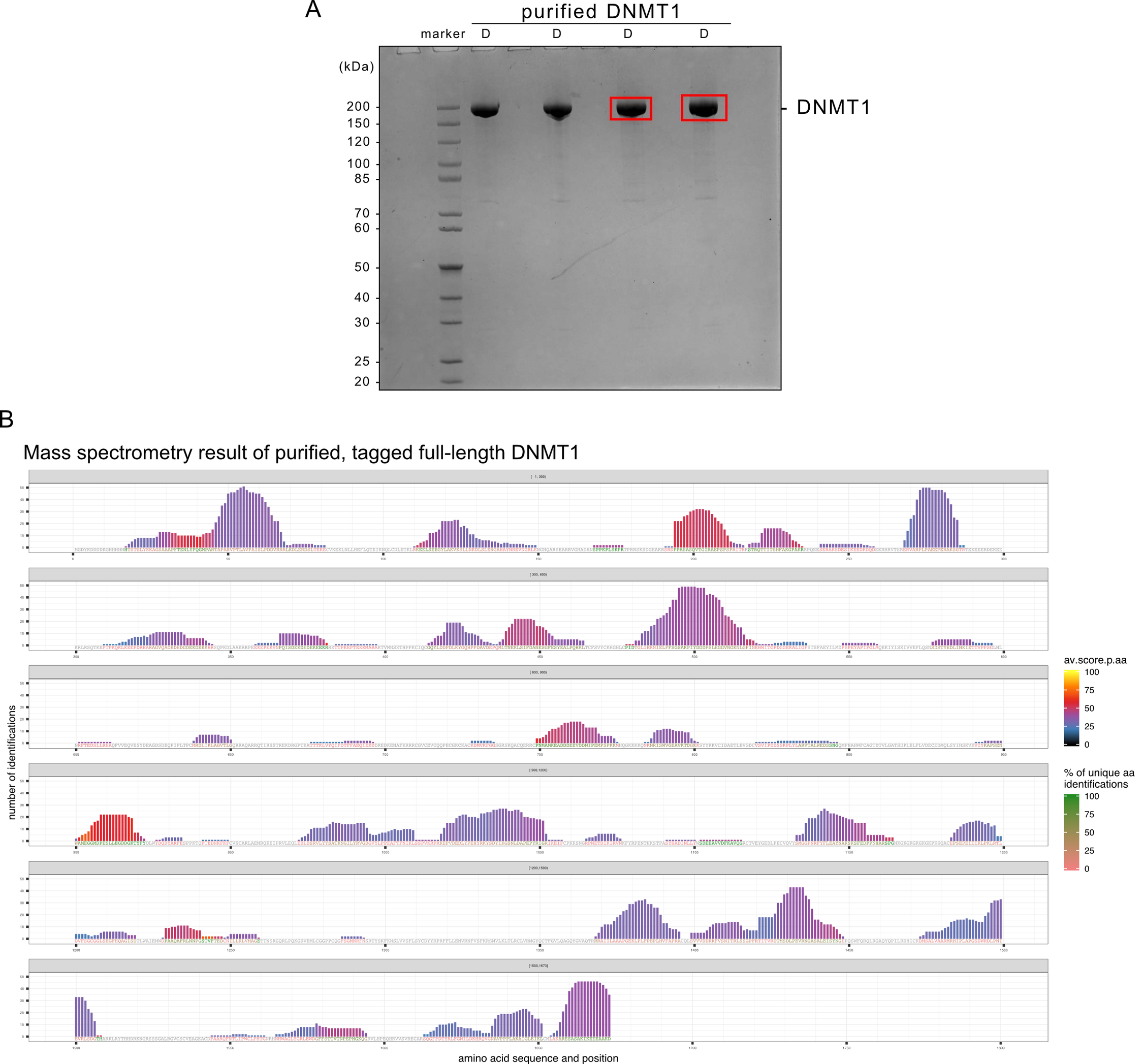

Supplement: S1 Fig — (A) Coomassie-stained SDS-PAGE gel of purified full-length DNMT1 at varying concentrations. The red boxes mark samples analyzed by mass spectrometry. (B) Mass spectrometry result using acid hydrolysis confirms the presence of full-length (N-terminally tagged) DNMT1 as indicated by the identified peptides over the full range of DNMT1. (TIF) [file pone.0307850.s001.tif]

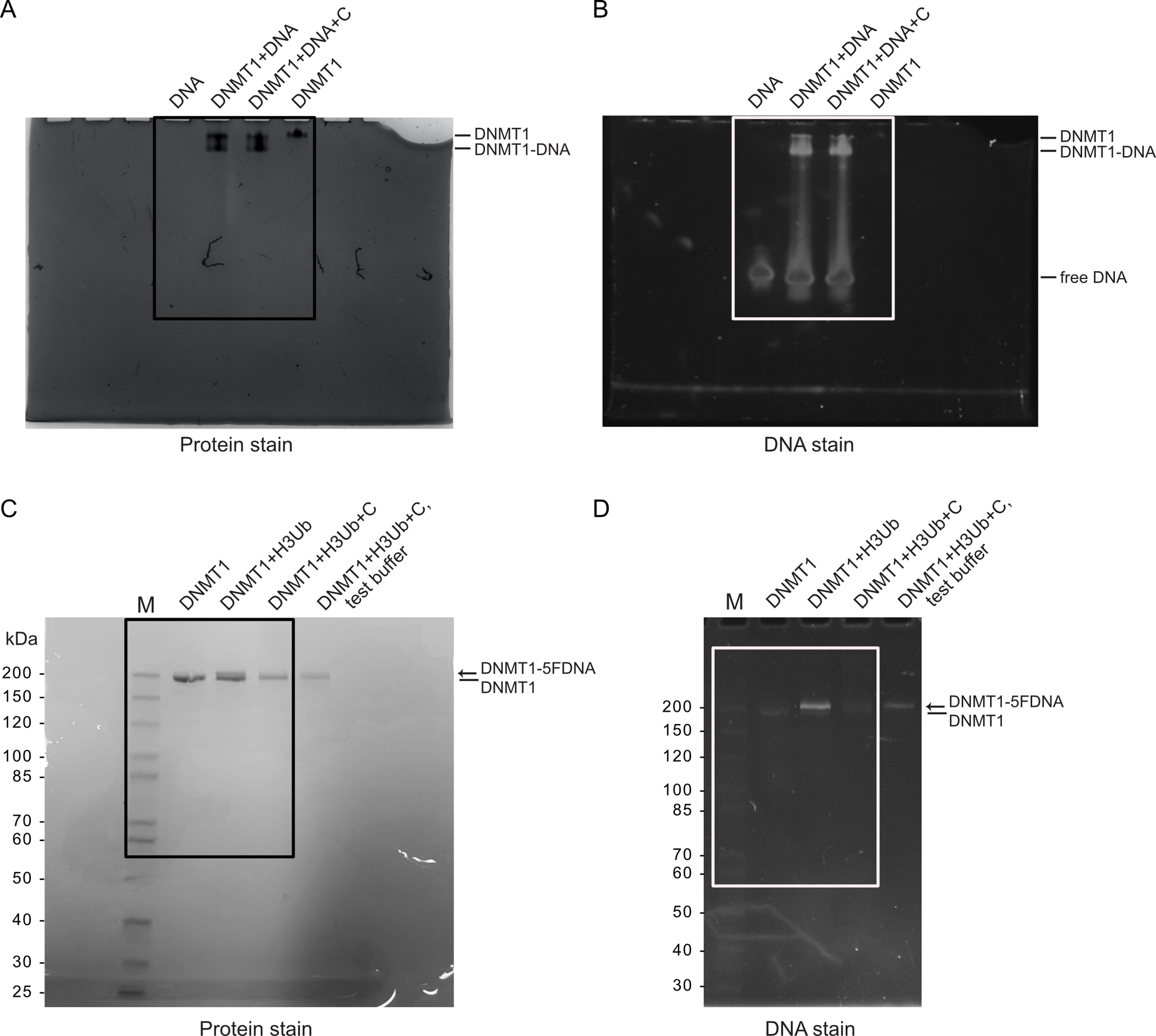

Supplement: S2 Fig — (A-B) Binding of DNMT1 to DNA in the presence and absence of GSK3852279B inhibitor (labeled as [C]) is analysed by native gel electrophoresis. The gel is stained to detect either protein (A) or DNA (B). (C-D) DNA methylation by DNMT1 in the presence of H3Ub2-peptide and GSK3852279B inhibitor (labeled as [C]) is analyzed by SDS-PAGE. The gel is stained to detect either protein (C) or DNA (D). Free DNMT1 and DNMT1 cross-linked to 5FDNA (DNMT1-5FDNA) are indicated. Boxes indicate the cropped area used in Fig 2. (TIF) [file pone.0307850.s002.tif]

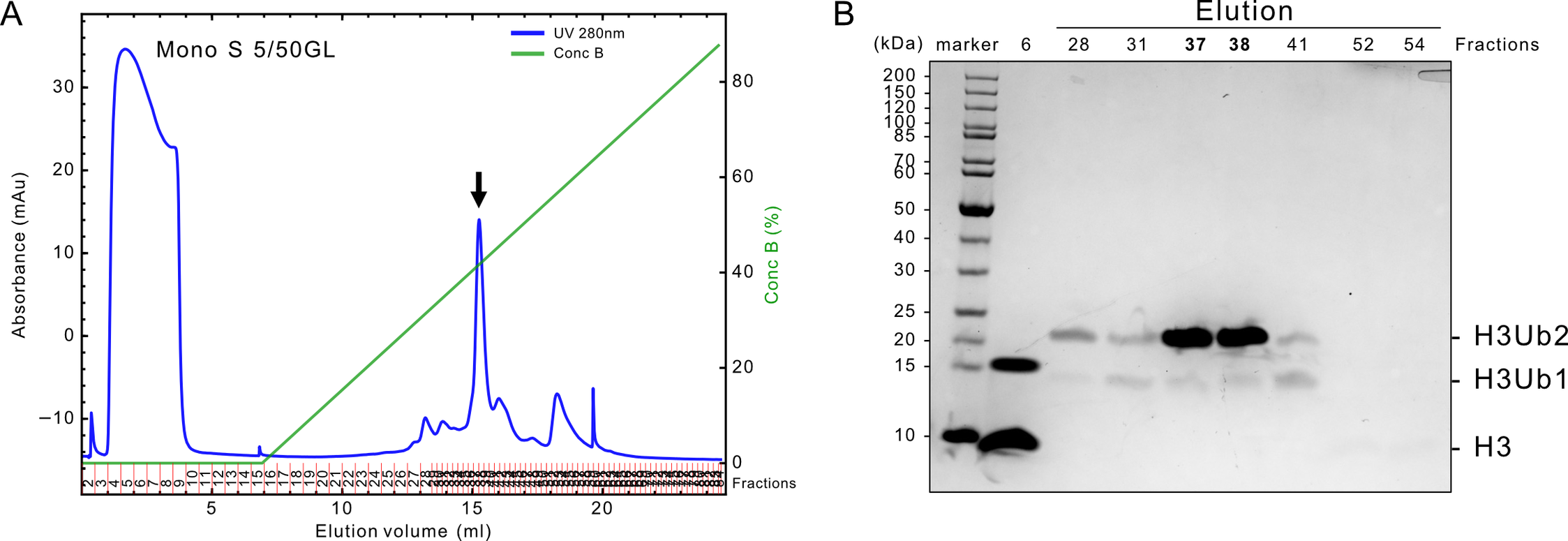

Supplement: S3 Fig — (A) H3Ub2-peptide was purified on a Mono S 5/50 GL chromatography column using a salt concentration gradient (concentration buffer B). (B) Fractions were analyzed by SDS-PAGE. The arrow and bold fraction numbers indicate the main peak of H3Ub2-peptide with >90% purity over mono-ubiquitinated (H3Ub1) or non-ubiquitinated (H3) species. (TIF) [file pone.0307850.s003.tif]

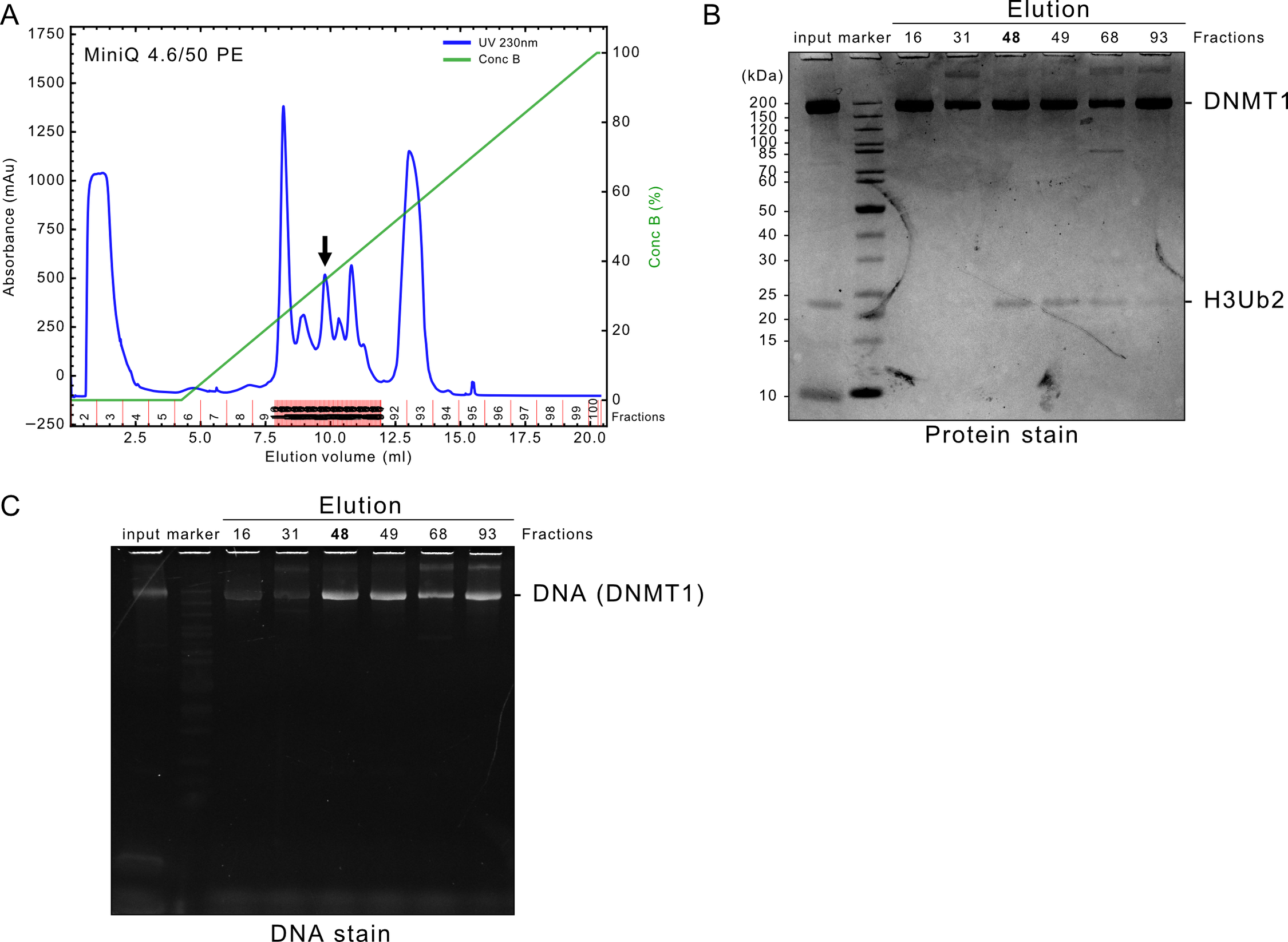

Supplement: S4 Fig — (A) The DNMT1:5FDNA: H3Ub2-peptide complex was purified using a MiniQ 4.6/50 PE chromatography column using a salt concentration gradient (concentration buffer B). Fractions were analyzed by SDS-PAGE using a protein stain (B) and a DNA stain (C). The arrow and bold fraction number indicate the main peak of the productive complex. (TIF) [file pone.0307850.s004.tif]
