# Supplementary material for: Structural insight into the DNMT1 reaction cycle by cryo-electron microscopy
Source: PLoS One. 2024 Sep 3;19(9):e0307850. doi: 10.1371/journal.pone.0307850 (PMC11371216; doi:10.1371/journal.pone.0307850)
Supplement: S1 Table — (DOCX) [file pone.0307850.s005.docx]

| **Species** | **PDB/EMDB entries** | **Short description** | **Residues** | **Resolution (Å)** | **Publication** |
| --- | --- | --- | --- | --- | --- |
| *H. sapiens* | 3EPZ | RFTS domain | 351-600 | 2.31 | Syeda *et al.* 2011 [38] |
|  | 3PTA | With DNA | 646-1600 | 3.60 | Song *et al.* 2011 [34] |
|  | 3SWR | With Sinefungin | 601-1600 | 2.49 | N/A |
|  | 4WXX | With SAH | 351-1600 | 2.62 | Zhang *et al.* 2015 [14] |
|  | 4YOC | With USP7/HAUSP complex | 600-1600 | 2.92 | Cheng *et al.* 2015 [44] |
|  | 4Z96, 4Z97 | With USP7 ± K1115Q mutant | 1098-1129 | 2.85, 3.00 | N/A |
|  | 5WVO | RFTS domain with K18/K23 ubiquitinated H3 | 351-600 | 2.00 | Ishiyama *et al.* 2017 [22] |
|  | 5YDR | RFTS domain with ubiquitin | 351-599 | 2.00 | Li *et al.* 2018 [23] |
|  | 6K3A | PCNA with DNMT1 PIP box motif | 161-180 | 2.30 | Jimenji *et al.* 2019 [12] |
|  | 6L1F | PHF20L1 Tudor1 with K142me1 DNMT1 | 140-145 | 1.90 | Lv *et al.* 2020 [45] |
|  | 6X9I, 6X9J, 6X9K | With Zebularine-containing 12mer dSDNA ± inhibitors | 729-1600 | 1.79-2.65 | Pappalardi *et al*. 2021 [6] |
|  | 7SFC, 7SFD, 7SFE, 7SFF, 7SFG | With Zebularine-containing 12mer dSDNA ± inhibitors/SAM | 729-1600 | 1.97-2.55 | Horton *et al*. 2022 [25] |
|  | 7XI9, 7XIB/ EMD-33200, EMD-33201 | Cryo-EM; With DNA and ubiquitinated H3 | 351-1616 | 2.52, 2.23 | Kikuchi *et al*. 2022 [24] |
|  | EMD-18418, EMD-50795, EMD-50801, EMD-50802 | Cryo-EM maps for apo DNMT1, DNMT1:productive DNA, DNMT1:H3Ub2-peptide, DNMT1:non-productive complexes | 1-1616^a^ | 3.3-6.0 | This study |
| *B. taurus* | 6PZV | RFTS domain with H3K9me3/ubiquitin | 349-594 | 3.01 | Ren *et al.* 2020 [42] |
|  | 7LMK, 7LMM | BAH1 domain with H4K20me3/2 | 725-897 | 2.65, 2.80 | Ren *et al*. 2021 [46] |
| *M. musculus* | 3AV4, 3AV5, 3AV6 | Apo; with AdoHcy; with AdoMet | 291-1620 | 2.75, 3.25, 3.09 | Takeshita *et al*. 2011 [15] |
|  | 3PT6, 3PT9 | With SAH ± DNA | 650/731-1602 | 3.00, 2.50 | Song *et al*. 2011 [34] |
|  | 4DA4 | With hm-CpG DNA | 731-1602 | 2.60 | Song *et al*. 2012 [9] |
|  | 5GUT, 5GUV | N1248A, R1279D mutants | 731-1602 | 2.10, 3.08 | Ye *et al*. 2018 [47] |
|  | 5WY1 | T1505A mutant | 291-1620 | 3.27 | Kanada *et al*. 2017 [48] |
|  | 6W8V, 6W8W | With ACG/CCG DNA | 731-1602 | 3.12, 3.00 | Adam *et al*. 2020 [49] |

**Additional References as listed in S1 Table**

44. Cheng J, Yang H, Fang J, Ma L, Gong R, Wang P, et al. Molecular mechanism for USP7-mediated DNMT1 stabilization by acetylation. Nat Commun. 2015;6:7023. Epub 2015/05/12. doi: 10.1038/ncomms8023. PubMed PMID: 25960197; PubMed Central PMCID: PMCPMC4432644.

45. Lv M, Gao J, Li M, Ma R, Li F, Liu Y, et al. Conformational Selection in Ligand Recognition by the First Tudor Domain of PHF20L1. J Phys Chem Lett. 2020;11(18):7932-8. Epub 2020/09/05. doi: 10.1021/acs.jpclett.0c02039. PubMed PMID: 32885980.

46. Ren W, Fan H, Grimm SA, Kim JJ, Li L, Guo Y, et al. DNMT1 reads heterochromatic H4K20me3 to reinforce LINE-1 DNA methylation. Nat Commun. 2021;12(1):2490. Epub 2021/05/05. doi: 10.1038/s41467-021-22665-4. PubMed PMID: 33941775; PubMed Central PMCID: PMCPMC8093215

47. Ye F, Kong X, Zhang H, Liu Y, Shao Z, Jin J, et al. Biochemical Studies and Molecular Dynamic Simulations Reveal the Molecular Basis of Conformational Changes in DNA Methyltransferase-1. ACS Chem Biol. 2018;13(3):772-81. Epub 2018/01/31. doi: 10.1021/acschembio.7b00890. PubMed PMID: 29381856; PubMed Central PMCID: PMCPMC6913882.

48. Kanada K, Takeshita K, Suetake I, Tajima S, Nakagawa A. Conserved threonine 1505 in the catalytic domain stabilizes mouse DNA methyltransferase 1. J Biochem. 2017;162(4):271-8. doi: 10.1093/jb/mvx024.

49. Adam S, Anteneh H, Hornisch M, Wagner V, Lu J, Radde NE, et al. DNA sequence-dependent activity and base flipping mechanisms of DNMT1 regulate genome-wide DNA methylation. Nat Commun. 2020;11(1):3723. Epub 2020/07/28. doi: 10.1038/s41467-020-17531-8. PubMed PMID: 32709850; PubMed Central PMCID: PMCPMC7381644.
